# The Role of Ribonuclease 1 and Ribonuclease Inhibitor 1 in Acute Kidney Injury after Open and Endovascular Thoracoabdominal Aortic Aneurysm Repair

**DOI:** 10.3390/jcm9103292

**Published:** 2020-10-14

**Authors:** Elisabeth Zechendorf, Alexander Gombert, Tanja Bülow, Nadine Frank, Christian Beckers, Arne Peine, Drosos Kotelis, Michael J. Jacobs, Gernot Marx, Lukas Martin

**Affiliations:** 1 Department of Intensive Care and Intermediate Care, University Hospital RWTH Aachen, 52062 Aachen, Germany; ezechendorf@ukaachen.de (E.Z.); nfrank@ukaachen.de (N.F.); cbeckers@ukaachen.de (C.B.); apeine@ukaachen.de (A.P.); gmarx@ukaachen.de (G.M.); 2 European Vascular Center Aachen-Maastricht, University Hospital RWTH Aachen, 52062 Aachen, Germany; agombert@ukaachen.de (A.G.); dkotelis@ukaachen.de (D.K.); mjacobs@ukaachen.de (M.J.J.); 3 Department of Medical Statistics, University Hospital RWTH Aachen, 52062 Aachen, Germany; tbuelow@ukaachen.de

**Keywords:** thoracoabdominal aortic aneurysm, ribonuclease, ribonuclease inhibitor 1, biomarker, complex aortic surgery, acute kidney injury

## Abstract

Acute kidney injury (AKI) is one of the most common post-operative complications and is closely associated with increased mortality after open and endovascular thoracoabdominal aortic aneurysm (TAAA) repair. Ribonuclease (RNase) 1 belongs to the group of antimicrobial peptides elevated in septic patients and indicates the prediction of two or more organ failures. The role of RNase 1 and its antagonist RNase inhibitor 1 (RNH1) after TAAA repair is unknown. In this study, we analyzed RNase 1 and RNH1 serum levels in patients undergoing open (*n* = 14) or endovascular (*n* = 19) TAAA repair to determine their association with post-operative AKI and in-hospital mortality. Increased RNH1 serum levels after open TAAA repair as compared with endovascular TAAA repair immediately after surgery and 12, 48, and 72 h after surgery (all *p* < 0.05) were observed. Additionally, elevated RNase 1 and RNH1 serum levels 12, 24, and 48 h after surgery were shown to be significantly associated with AKI (all *p* < 0.05). RNH1 serum levels before and RNase 1 serum levels 12 h after TAAA repair were significantly correlated with in-hospital mortality (both *p* < 0.05). On the basis of these findings, RNase 1 and RNH1 may be therapeutically relevant and may represent biomarkers for post-operative AKI and in-hospital mortality.

## 1. Introduction

Thoracoabdominal aortic surgery is associated with several post-operative complications and increased mortality [1]. Mortality is 8.3% 30 days after open surgery and 5.8% after endovascular surgery [2]. Multiple organ failure is one of the dreaded post-operative complications after open and endovascular surgical treatment of thoracoabdominal aortic aneurysms (TAAA). Furthermore, acute kidney injury (AKI) is one of the most common organ failures after TAAA repair with an incidence between 13% and 42%. In addition to cardiovascular morbidity, AKI is associated with increased mortality [3,4,5]. AKI is diagnosed according to the Kidney Disease – Improving Global Outcomes (KDIGO) criteria. According to KDIGO, AKI is diagnosed when serum creatinine increases by ≥0.3 mg/dl (26.5 μmol/l) within 48 h or ≥1.5-fold, or when there is a reduction in urine volume to <0.5 mL/kg/h over 6 h. The three stages of AKI (I–III) are based on the aforementioned criteria: In addition to urinary excretion, diagnosis of AKI is based on patient serum creatinine levels. However, serum creatinine is a controversial biomarker for the detection of impaired renal function due to its delayed increases and low sensitivity [6,7,8]. Therefore, the establishment of new clinically available and reliable biomarkers and therapeutic approaches for the treatment of AKI is necessary [9].

Ribonuclease (RNase) 1 is a host defense peptide of the innate immune system which is expressed ubiquitously in various tissues and body fluids [10]. The primary function of RNase 1 is the degradation of circulating double- and single-stranded RNAs [10]. In a previous study, we observed increased RNase 1 serum levels in septic patients as compared with healthy subjects. Furthermore, we demonstrated that RNase 1 serum concentrations indicated a prediction of dysfunction of two or more organs in septic patients [11]. RNase inhibitor 1 (RNH1) is also ubiquitously expressed as an inhibitor of RNase 1 in a variety of tissues that inhibits its activity by direct binding [12,13]. In a recent study, we detected increased RNH1 serum levels in septic patients, as well as elevated extracellular RNA [14]. However, the role of RNase 1 and RNH1 as biomarkers of AKI and in-hospital mortality in the setting of TAAA repair has not yet been investigated.

The aim of this study was to evaluate the role of RNase 1 and RNH1 as potential biomarkers, as well as therapeutic strategies for the prediction of post-operative AKI and in-hospital mortality in patients undergoing complex open and endovascular TAAA repair.

## 2. Experimental Section

### 2.1. Study Approval and Design

**Figure 1:**
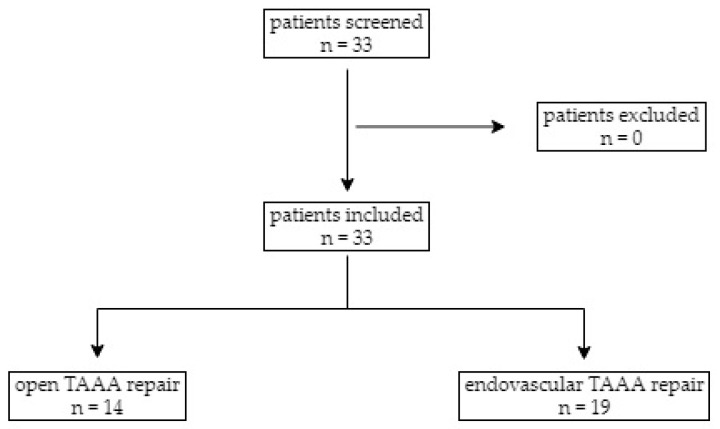
Screening and exclusion of patients.

All serum samples were collected, in 2017, between January and December after approval by the internal ethics committee of the University Hospital Aachen (EK004/14). Written informed consent was obtained preoperatively from all subjects. After screening and exclusion of patients who met the exclusion criteria, including emergency procedures, age below 18 years, pregnancy, chronic kidney disease requiring permanent dialysis treatment, and ongoing immunosuppressive therapy, 33 patients were included in this study. Of these, 14 patients underwent open repair and 19 patients underwent endovascular TAAA repair (Figure 1). Demographic data, medical history, and daily physiological variables were obtained from patient records and electronic flowcharts at the bedside (IntelliSpace Critical Care and Anesthesia, Philips Healthcare, Andover, MA, USA). On the basis of serum creatinine levels and 24-h urine output detection during the first 72 h after surgery, AKI was defined according to the KDIGO criteria [9]. The trial is registered under the ClinicalTrials.gov number NCT03093857. The cohort of patients has been previously described in a recently published study [15].

### 2.2. Surgery

During open TAAA repair, different techniques were used to reduce intraoperative organ ischemia. Sequential aortic clamping, extracorporal circulation using distal aortic perfusion, and selective visceral perfusion are established methods to reduce organ damage during surgery [16,17] [18]. Renal perfusion was achieved using 4 °C tempered Custodiol^®^ (Dr. Franz Köhler Chemie, Bensheim, Germany) [19]. In the case of endovascular TAAA repair, a contrast agent was carefully applied to avoid kidney failure, leading to a mean application of 65 ± 17 mL per endovascular procedure [20].

### 2.3. Serum Sampling

Serum samples were collected after patients were enrolled in the study at six different time points (before surgery, after admission to the intensive care unit (ICU), and 12, 24, 48, and 72 h after surgery). Serum samples were centrifuged at 3000 rpm for 10 min at room temperature after 10 min of coagulation. Samples were stored at −80 °C until RNase 1 and RNH1 serum levels were measured.

### 2.4. Human RNase Inhibitor 1 (RNH1) Enzyme-Linked Immunosorbent Assay

RNH1 serum levels were assessed using an ELISA designed by our research group. A 96-well plate was coated with 100 µL of diluted (2.5 µg/mL in PBS) capture antibody (#ABIN1342154, Abnova, Taipeh, Taiwan) and incubated at 4 °C overnight. After washing with 0.05% Tween in PBS for 3 min three times, the plate was blocked in blocking buffer containing 5% fat free milk and 10% HS for two h at room temperature. A standard series was prepared in a range from 0.78 to 100 ng/mL using a recombinant RNH1 Protein (#ABIN1318405, Abnova). After repeating the wash step, 100 µL of standard and samples were added to each well and incubated for 2 h at room temperature. Wells were subsequently aspirated and washed five times, followed by addition of 100 µL of diluted detection antibody with a working concentration of 1 µg/mL to each well. Next, the wells were aspirated, washed, and incubated, for 1 h, in 100 µL of an HRP-conjugated goat anti-rabbit antibody (#31460, Thermo Fisher Scientific, Bedford, MA, USA). The wash step was repeated, and the TMB substrate solution was added and incubated, for 5 to 20 min, at room temperature protected from light before the reaction was stopped with 2 N sulfuric acid. The optical density was determined at a wavelength of 450 nm using a plate reader (Tecan Group, Männedorf, Switzerland). For statistical analysis, GraphPad 7 (GraphPad Inc., San Diego, CA, USA) was used.

### 2.5. Human Ribonuclease (RNase) 1 Enzyme-Linked Immunosorbent Assay

Levels of RNase 1 in human serum were determined using a commercial ELISA kit (#SEK13468, Sino Biological Inc., Peking, China) according to the manufacturer’s instructions. For analysis, the optical density was measured at 450 nm using a microplate reader (Tecan).

### 2.6. Endpoints

To investigate the role of RNase 1 and RNH1 in AKI after TAAA repair, especially to analyze differences between open and endovascular TAAA repair, we examined preoperative renal function in relation to RNase 1 and RNH1 serum levels. In order to analyze only preoperative kidney function, patients with preexisting kidney disease (defined as preoperative serum creatinine >1.25 mg/dL according to the cut-off used in the Cleveland Clinic foundation score [21]) were excluded. Furthermore, we investigated the relationship between RNase 1 and RNH1 serum levels and in-hospital mortality in patients undergoing open and endovascular TAAA repair, as well as the differences in these two surgical techniques.

The association of serum levels of RNase 1 and RNH1 was also investigated with post-operative endpoints, such as ICU stay, sepsis and inflammatory markers C-reactive protein (CRP), procalcitonin (PCT), and interleukin-6 (IL-6). Sepsis is defined as a life-threatening organ dysfunction that is identified by a 2-point increase in SOFA score (sequential (sepsis-related) organ failure assessment) [22].

### 2.7. Statistical Analysis

Continuous data are presented as box-whisker plots. Lines inside the boxes represent the median and the pluses represent the mean. The box is defined by Q1 and Q3. The whiskers range from Q1 to Q1 + 1.5 ∗ (Q3 − Q1) and Q3 to Q3 − 1.5 ∗ (Q3 − Q1), with observations outside of the whiskers shown as points classified as outliers. Categorical data are presented as absolute frequencies and percentages. RNase 1 and RNH1 serum levels are visualized over time again as box-whisker plots. To compare patient characteristics between open TAAA repair and endovascular TAAA repair, unpaired t-tests were used. For each point in time, a univariable logistic regression model was applied to assess the association between the outcome variables AKI (yes/no) and in-hospital mortality (died/survived) with RNase 1 and RNH1 serum levels.

The diagnostic quality of RNase 1 and RNH1 serum levels with respect to AKI and in-hospital mortality was evaluated for each point in time using the receiver operating characteristic (ROC) analysis. Sensitivity (Se), specificity (Sp), positive and negative likelihood ratio (LR+ and LR−), area under the curve (AUC), and the optimal cut-off value according to the Youden index were given in addition to the ROC curves for each time point and the respective diagnostic variable.

A monotone correlation between RNase 1 and RNH1 and perioperative variables was evaluated using Spearman correlation coefficient. Due to the various sample sizes, the reliability of the correlation coefficient was assessed through the p-value testing for a correlation coefficient different from 0. For all Spearman correlation coefficients statistically significant different from 0, the respective *p*-value has been stated. A Spearman correlation coefficient above 0.3 is considered to be an indicator for a moderate correlation, and above 0.5 to be a strong monotone correlation.

The level of significance was set at 5%. No adjustments were made for multiple comparisons due to the exploratory nature of this study. Statistical analyses were performed using SAS software version 9.4 (SAS Institute, Cary, NC, USA) and R, version 3.6.1. ROC analysis was performed using MedCalc, version 19.2.5.

## 3. Results

### 3.1. Study Population

The median age of patients undergoing endovascular TAAA repair (74 (69–78)) was significantly higher than those undergoing open TAAA repair (51 (37–65)) (*p* < 0.001). Pre-operative renal insufficiency was observed in three patients undergoing open (21.4%) and in two patients undergoing endovascular TAAA repair (10.5%). Ten patients undergoing open (71.4%) and seven (36.8%) undergoing endovascular TAAA repair developed post-operative AKI as diagnosed according to the KDIGO classification criteria. All details of patient characteristics can be found in Table 1.

### 3.2. Ribonuclease (RNase) 1 Serum Levels

**Figure 2:**
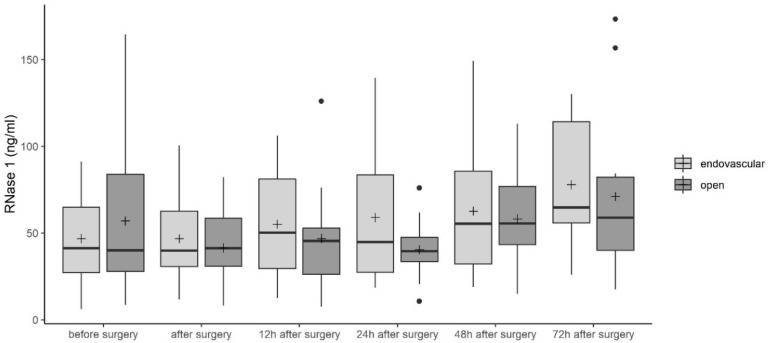
Ribonuclease (RNase) 1 serum levels in patients undergoing open or endovascular thoracoabdominal aortic aneurysm (TAAA) repair. Data are presented as box-whisker plots. Lines inside the boxes represent the median and the pluses represent the mean. The box is defined by Q1 and Q3. The whiskers range from Q1 to Q1 + 1.5 ∗ (Q3 − Q1) and Q3 to Q3 − 1.5 ∗ (Q3 − Q1), with observations outside of the whiskers shown as points classified as outliers. Unpaired t-test (two-tailed) was used for statistical analysis of the two groups. RNase 1, ribonuclease 1.

We first investigated serum levels of RNase 1 in patients undergoing open or endovascular TAAA repair. RNase 1 concentrations decreased in patients undergoing open TAAA repair from 56.9 ± 44.4 ng/mL before surgery to 40.4 ± 17.5 ng/mL 24 h after surgery (Figure 2). Forty-eight hours after surgery, RNase 1 serum levels increased so that a concentration of 71.1 ± 45.9 ng/mL was reached 72 h after surgery (Figure 2). In patients undergoing endovascular TAAA repair, RNase 1 serum levels increased over time from 46.78 ± 26.7 ng/mL to 77.86 ± 37.2 ng/mL (Figure 2). When comparing the two groups, no significant difference was detected (Figure 2).

### 3.3. RNase Inhibitor 1 (RNH1) Serum Levels

**Figure 3:**
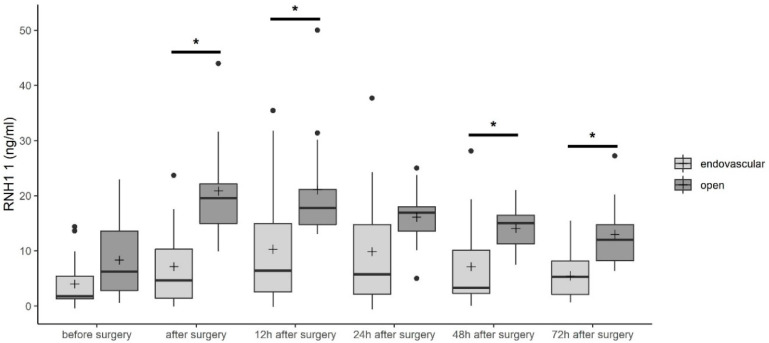
RNase inhibitor 1 (RNH1) serum levels in patients undergoing open or endovascular TAAA repair. Data are presented as box-whisker plots. Lines inside the boxes represent the median and the pluses represent the mean. The box is defined by Q1 and Q3. The whiskers range from Q1 to Q1 + 1.5 ∗ (Q3 − Q1) and Q3 to Q3 − 1.5 ∗ (Q3 − Q1), with observations outside of the whiskers shown as points classified as outliers. Unpaired *t*-test (two-tailed) was used for statistical analysis of the two groups. * *p* < 0.05; RNH1, RNase inhibitor 1.

As described before, RNH1 is an antagonist of RNase 1 and inhibits its enzymatic activity by direct binding. Therefore, we next investigated serum levels of RNH1 in patients undergoing TAAA repair. In contrast to RNase 1 serum concentrations, we detected increased RNH1 concentrations from 8.3 ± 6.9 ng/mL and 4.2 ± 4.5 ng/mL before open and endovascular TAAA repair to 18.9 ± 5.9 ng/mL and 11.5 ± 10.3 ng/mL, respectively, 12 h after surgery (Figure 3). Afterwards, RNH1 serum concentrations decreased to 13.0 ± 7.3 ng/mL and 5.5 ± 4.3 ng/mL 72 h after open and endovascular TAAA repair, respectively (Figure 3). Before surgery, no significant differences between the two groups were observed. After surgery and 12, 48, and 72 h after admission to the ICU, significantly increased RNH1 serum levels were observed after open TAAA repair as compared with endovascular TAAA repair (all *p* < 0.05, Figure 3). 

### 3.4. Correlation of RNase 1 and RNH1 with Acute Kidney Injury (AKI)

**Figure 4:**
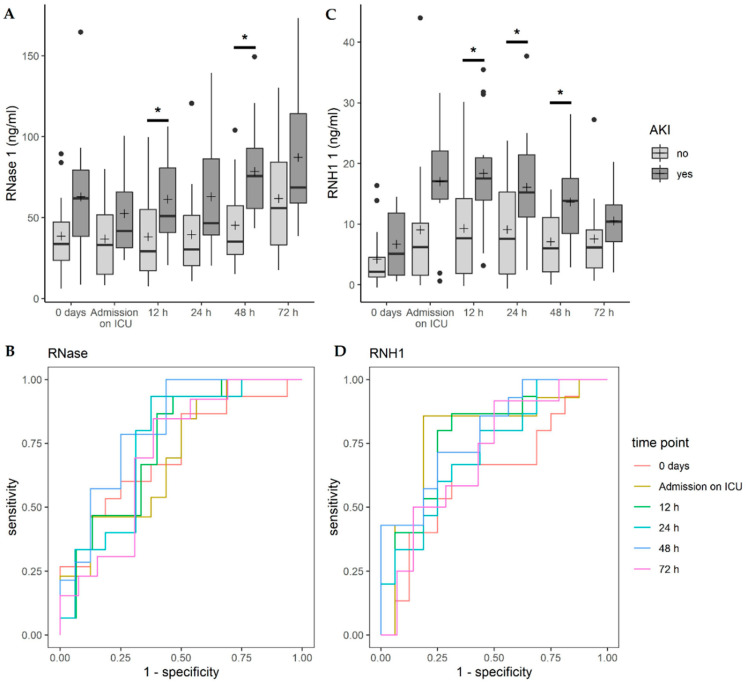
The correlation of (**A**) RNase 1 and (**C**) RNH1 with acute kidney injury (AKI). Data are presented as box-whisker plots. Lines inside the boxes represent the median and the pluses represent the mean. The box is defined by Q1 and Q3. The whiskers range from Q1 to Q1 + 1.5 ∗ (Q3 − Q1) and Q3 to Q3 − 1.5 ∗ (Q3 − Q1), with observations outside of the whiskers shown as points classified as outliers. ROC analysis of the diagnostic accuracy of (**B**) RNase 1 and (**D**) RNH1 serum levels for acute kidney injury in patients undergoing endovascular or open TAAA repair. * *p* < 0.05; RNase, ribonuclease; RNH1, RNase inhibitor 1.

Analyzing the effect from RNase 1 and RNH1 serum levels on the probability of suffering AKI with a univariable logistic regression model for each point in time, RNase 1 showed a statistically significant effect 12 h after surgery (*p* = 0.0327, OR = 1.035) and 48 h after surgery (*p* = 0.0144, OR = 1.045, Figure 4A). Higher RNH1 serum levels conveyed a statistically significant higher probability of experiencing AKI 12 h after surgery (*p* = 0.0199, OR = 1.129), 24 h after surgery (*p* = 0.0435, OR = 1.106), and 48 h after surgery (*p* = 0.0194, OR = 1.178, Figure 4C).

Regarding a correlation between RNase 1 and AKI, focusing on all patients suffering from post-operative AKI according to the KDIGO classification, a test accuracy of 0.702–0.750 was observed (Figure 4B). From 24 h to 48 h after surgery, the sensitivity reached 93.33–100%, and the specificity was 56.25–62.50% (Figure 4B). RNH1 showed good test accuracy for post-operative AKI with an AUC between 0.702 and 0.790 for all time points after surgery (Figure 4C). Upon admission to the ICU, the test accuracy was 0.781, with a sensitivity of 85.71% and a specificity of 81.25%. Further details can be found in the Supplementary Materials Tables S1 and S2.

While assessing the predictive abilities of RNase 1 and RNH1 for AKI separated in KDIGO 0 (n = 16) and 3 (n = 4), a favorable test accuracy for RNase 1 measured 48 h after TAAA surgery was observed (AUC 0.969, sensitivity 100%, specificity 87.5%, Table 2).

### 3.5. Correlation of RNase 1 and RNH1 with In-Hospital Mortality

**Figure 5:**
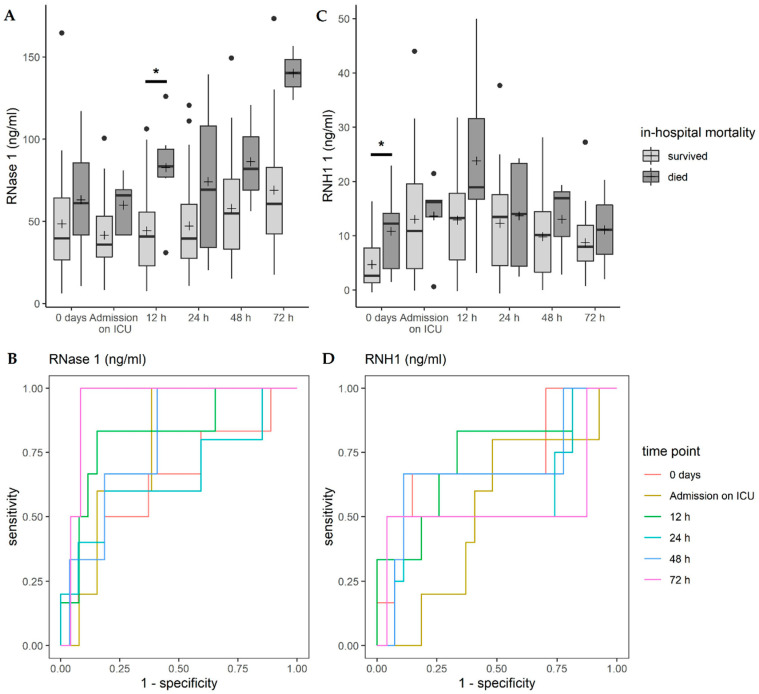
The correlation of (**A**) RNase 1 and (**C**) RNH1 with in-hospital mortality. Data are presented as box-whisker plots. Lines inside the boxes represent the median and the pluses represent the mean. The box is defined by Q1 and Q3. The whiskers range from Q1 to Q1 + 1.5 ∗ (Q3 − Q1) and Q3 to Q3 − 1.5 ∗ (Q3 − Q1), with observations outside of the whiskers shown as points classified as outliers. ROC analysis of the diagnostic accuracy of (**B**) RNase 1 and (**D**) RNH1 serum levels for in-hospital mortality in patients undergoing endovascular or open TAAA repair. * *p* < 0.05; RNase, ribonuclease; RNH1, RNase inhibitor 1.

Analyzing the effect of RNase 1 and RNH1 serum levels on in-hospital mortality with a univariable logistic regression model for each point in time, RNase 1 showed a statistically significant effect 12 h after surgery (*p* = 0.018, OR = 1.048, Figure 5A). For higher RNH1 serum levels, a statistically significant higher probability to die was observed at 0 days (*p* = 0.0414, OR = 1.174, Figure 5C).

The test accuracy of RNase 1 to predict in-hospital mortality increased over time, reaching 0.938 72 h after surgery, with a sensitivity of 100% and a specificity of 91.67% (Figure 5B). A moderate predictive accuracy for in-hospital mortality and RNH1 was also observed (Figure 5D). All details can be found in Tables S3 and S4.

### 3.6. Correlation of RNase 1 and RNH1 with Perioperative Variables

A Spearman correlation statistically significant different from 0 of RNase 1 and SOFA-Score was observed 48 and 72 h after surgery (*p* = 0.02 and 0.03, respectively), indicating a strong monotone correlation. Regarding the length of stay in the ICU, a statistically significant correlation different from 0 was observed for RNase 1 levels 48 h after surgery (*p* = 0.01), indicating a moderate monotone correlation. Statistically significant correlations different from 0 for several time points of RNH1 measurement and all assessed parameters were observed. Further correlations of RNase 1 and RNH1 with variables are shown in Table 3 and Table 4. Although other than the pointed correlation coefficients of RNase 1 and RNH1 indicated moderate or strong correlations, for example, RNase 1 and PCT before surgery, the sample size for these markers were insufficient to make reliable conclusions about the magnitude of the correlation. 

## 4. Discussion

Thoracoabdominal aortic surgery is associated with post-operative complications and increased mortality [1]. Multiple organ failure is one of the dreaded post-operative complications after open and endovascular surgical treatment of thoracoabdominal aortic aneurysms (TAAA). AKI is one of the feared post-operative complications. Due to the absence of reliable biomarkers, such as serum creatinine, the establishment of new clinically available and reliable biomarkers and therapeutic approaches for the treatment of AKI is essential. In this study, we measured the RNase I and RNH1 levels in open and endovascular TAAA repair to determine their association with post-operative AKI. We showed, for the first time, that RNase 1 and its antagonist RNH1 play a role in open and endovascular TAAA repair and are associated with post-operative AKI and in-hospital mortality.

Thoracoabdominal aortic surgery is associated with a high mortality rate; the literature describes a mortality of 8.3% 30 days after open surgery and 5.8% after endovascular surgery [2]. In this study population, we detected a higher mortality rate of 14.3% after open surgery and 21.1% after endovascular surgery (Table 1), due to the fact that Greenberg and colleagues had examined a larger patient population. They analyzed samples from more than five years and in total they included 724 patients [2]. We investigated a collective of only 33 patients (Table 1). Furthermore, the number of patients with a type 2 TAAA was relatively high for this small study and urgent cases were also included in this study.

As a consequence of TAAA repair, open surgery may be related to an increased rate of organ damage, while endovascular TAAA repair leads to post-implantation syndrome and severe endothelial damage, both of which result in release of danger-associated molecular patterns (DAMPs). Extracellular RNA (eRNA) represents one of these DAMPs. Extracellular RNA binds to both TLR 3 and 7, inducing increased production of pro-inflammatory cytokines, such as tumor necrosis factor alpha, by translocation of nuclear factor kappa B [23,24]. This leads to an increased inflammatory reaction and results in organ dysfunction, such as AKI. Zhou and Yang described the involvement of eRNA in kidney failure [25]. RNase 1 recognizes pathogenic RNA and degrades it [10]. Therefore, increased release of RNase 1 after TAAA repair is expected. Indeed, we detected increased RNase 1 in the serum of patients after TAAA repair up to 72 h after surgery (Figure 2). An antagonist of RNase 1, RNH1 protects the cytosolic compartments from the toxic effects of RNases, however, this also has the consequence of inhibiting their antimicrobial properties [26]. In previous studies by this group, we also detected increased RNH1 and RNase 1 levels in serum of septic patients as compared with healthy subjects [11,14]. In line with these findings, we observed increased RNH1 serum levels after open and endovascular TAAA repair (Figure 3). After open TAAA repair, increased RNH1 serum levels were detected as compared with serum levels after endovascular TAAA repair (Figure 3). However, for RNase 1 serum levels, there was no difference between open and endovascular TAAA repair (Figure 2). 

Martin et al. reported that elevated RNase 1 serum levels in patients with sepsis indicated dysfunction of two or more organs [11]. In line with Martin et al., we demonstrated that patients with increased RNase 1 serum levels, 12 and 48 h after surgery, exhibited a higher probability of suffering AKI (Figure 4). Additionally, Martin et al. demonstrated that patients with renal dysfunction experienced significantly higher RNase 1 levels as compared with those without renal dysfunction [11]. Indeed, we also found that patients with higher RNase 1 serum levels, 48 h after surgery, suffered a higher probability of stage 3 than stage 1 AKI (Table 2). Furthermore, we demonstrated that patients with higher RNH1 serum levels, 12, 24, and 48 h after surgery, also exhibited a higher probability of suffering AKI (Figure 4). Twelve hours after TAAA repair, we observed that RNase 1 serum levels correlated with in-hospital mortality (Figure 5). This may be due to the fact that the highest mean RNH1 serum levels were measured 12 h after surgery (15.34 ± 11.29). On the basis of these findings, it can be assumed that RNase 1 is strongly inhibited by RNH1 at this time, which means that RNase 1 is unable to cleave eRNA. This results in an increased inflammatory response and may be associated with organ dysfunction, which may explain the increased mortality at this time point.

Next, we investigated the correlation of RNase 1 and RNH1 with perioperative variables. We found, for the first time, that RNase 1 serum levels had a strong monotone correlation with 48 and 72 h time points after surgery, and RNH1 serum levels had a moderate monotone correlation 24 h after surgery with SOFA score (Table 3 and Table 4). The length of stay in the ICU had a moderate monotone correlation with RNase 1 serum levels 48 h after surgery and with RNH1 serum levels 48 and 72 h after surgery (Table 3 and Table 4). On the basis of these findings, RNase 1 and RNH1 serum levels may have the potential to predict post-operative sepsis, and thus the length of a hospital stay. 

## 5. Limitation and Conclusions

Since our investigation is limited to a small patient collective with a variation in the number of measurements of some variables, further investigations with a larger collective must be aimed at to underline our results. Moreover, our measurements were limited to RNase 1 and RNH1 serum levels, the analysis of eRNA concentrations in serum would be helpful to confirm the findings of this study.

In conclusion, our data show, for the first time, that open TAAA repair results in significantly increased RNH1 serum levels as compared with endovascular TAAA repair, after admission to the ICU and 12, 48, and 72 h after surgery. We found that RNase 1 serum levels 12 and 48 h after surgery and RNH1 serum levels 12, 24 and 48 h after surgery showed a statistically significantly higher probability of suffering AKI. Furthermore, we demonstrated, for the first time, that RNH1 exhibits good test accuracy for post-operative AKI. In addition, higher RNase 1 serum levels 12 h after surgery and increased RNH1 serum levels on day 0 convey a significantly higher probability for in-hospital mortality. On the basis of these findings, RNase 1 and RNH1 may be therapeutically important and may represent biomarkers for post-operative AKI and in-hospital mortality.

## Figures and Tables

**Table 1:** Patient characteristics.

	Open TAAA Repair (n = 14)	Endovascular TAAA Repair (n = 19)	*p*-Value
Age (year) (IQR)	51 (37–65)	74 (69–78)	<0.001
Male sex (%)	8 (57.1)	9 (47.4)	0.593
BMI (kg/m^2^) (IQR)	25.7 (20.6–30.6)	25.4 (21.5–27.1)	0.600
Diabetes mellitus (%)	2 (14.3)	4 (21.1)	0.685
Smoker (%)	4 (28.6)	8 (42.1)	0.440
Operation time (min) (IQR)	312.5 (295.5–474.5)	392.0 (280.0-460.0)	0.908
LOS ICU (days) (IQR)	5 (4–21)	3 (6–2)	0.075
LOS in-hospital (days) (IQR)	27 (19–38)	15 (9–35)	0.190
In-hospital mortality (%)	2 (14.3)	4 (21.1)	0.631
Pre-operative renal insufficiency (%)	3 (21.4)	2 (10.5)	0.4039
Post-operative acute kidney injury (%)	10 (71.4)	7 (36.8)	0.051

Data are presented as n (%) or median (IQR). Unpaired t-test (two-tailed) was used for statistical analysis. IQR, interquartile ranges (Q1–Q3); BMI, body mass index; LOS, length of stay; ICU, intensive care unit.

**Table 2:** RNASE to predict AKI (stadium = 0) vs. AKI (stadium = 3).

Time of Measurement	Optimal Cut-Off (Youden Index)					AUC
	Cut-Off (ng/mL)	Sensitivity (%)	Specificity (%)	LR+	LR−	
0 days n = 20	≥57.64	75.00 [19.4, 99.4]	81.25 [54.4, 96.0]	4.00	0.31	0.828 [0.595, 0.957]
Admission on ICU n = 20	≥31.00	100.00 [39.8, 100.0]	50.00 [24.7, 75.3]	2.00	-	0.672 [0.429, 0862]
12 h n = 19	≥61.42	75.00 [19.4, 99.4]	86.67 [59.5, 98.3]	5.63	0.29	0.817 [0.575, 0.954]
24 h n = 20	≥32.81	100.00 [39.8, 100.0]	62.50 [35.4, 84.8]	2.67	-	0.797 [0.560, 0.941]
48 h n = 20	≥67.81	100.00 [39.8, 100.0]	87.50 [61.7, 98.4]	8.00	-	0.969 [0.779, 1.000]
72 h n = 17	≥62.28	100.00 [39.8, 100.0]	69.23 [38.6, 90.9]	3.25	-	0.904 [0.663, 0.992]

**Table 3:** Spearman’s correlation coefficient for RNase 1 and perioperative variables. The number of included patients is shown below the coefficient. If the coefficient was statistically significantly different from 0, a *p*-value is stated below the number.

	Before Surgery	After Surgery	12 h After Surgery	24 h After Surgery	48 h After Surgery	72 h After Surgery
SOFA Score	x	0.20292	0.22965	0.25394	0.57451	0.73193
		(n = 6)	(n = 31)	(n = 29)	(n = 15)	(n = 8)
					(*p* = 0.0251)	(*p* = 0.0390)
Leucocytes	−0.03862	0.14354	−0.07718	−0.02492	0.37221	0.40294
	(n = 33)	(n = 30)	(n = 32)	(n = 28)	(n = 22)	(n = 16)
PCT	−0.86603	0.00000	0.20843	0.24527	0.33636	0.26190
	(n = 3)	(n = 9)	(n = 20)	(n = 24)	(n = 11)	(n = 8)
CRP	0.22086	0.10000	0.00351	−0.01342	0.01072	0.20000
	(n = 32)	(n = 9)	(n = 19)	(n = 18)	(n = 15)	(n = 15)
IL-6		−0.08571	0.54545	0.00000	0.54286	
	(n = 1)	(n = 6)	(n = 12)	(n = 12)	(n = 6)	(n = 1)
LOS ICU	0.04169	0.11124	0.05597	0.22260	0.46103	0.36988
	(n = 33)	(n = 31)	(n = 32)	(n = 32)	(n = 30)	(n = 26)
					(*p* = 0.0103)	

SOFA, sequential (sepsis-related) organ failure assessment; PCT, procalcitonin; CRP, C-reactive protein; IL, interleukin; LOS, length of stay; ICU, intensive care unit.

**Table 4:** Spearman’s correlation coefficient for RNH1 and perioperative variables. The number of included patients is shown below the coefficient. If the coefficient was statistically significantly different from 0, a *p*-value is stated below the number.

	Before Surgery	After Surgery	12 h After Surgery	24 h After Surgery	48 h After Surgery	72 h After Surgery
SOFA Score	x	−0.72471	0.31193	0.41576	0.32676	−0.11119
		(n = 6)	(n = 32)	(n = 29)	(n = 15)	(n = 7)
				(*p* = 0.0249)		
Leukocytes	−0.03996	−0.15045	−0.32879	−0.36971	−0.04123	0.27857
	(n = 33)	(n = 31)	(n = 33)	(n = 28)	(n = 22)	(n = 15)
PCT	0.86603	0.69457	0.41986	0.52142	0.60000	0.60714
	(n = 3)	(n = 9)	(n = 20)	(n = 24)	(n = 11)	(n = 7)
		(*p* = 0.0379)		(*p* = 0.0090)		
CRP	−0.05668	−0.13333	−0.43333	0.23220	0.54334	0.43736
	(n = 32)	(n = 9)	(n = 19)	(n = 18)	(n = 15)	(n = 14)
					(*p* = 0.0363)	
IL-6		0.54286	0.23776	0.75524	0.08571	
	(n = 1)	(n = 6)	(n = 12)	(n = 12)	(n = 6)	(n = 1)
				(*p* = 0.0045)		
LOS ICU	0.20422	0.18342	0.20371	0.21947	0.40419	0.44018
	(n = 33)	(n = 32)	(n = 33)	(n = 31)	(n = 30)	(n = 26)
					(*p* = 0.0267)	(*p* = 0.0244)

SOFA, sequential (sepsis-related) organ failure assessment; PCT, procalcitonin; CRP, C-reactive protein; IL, interleukin; LOS, length of stay; ICU, intensive care unit.

## Supplementary Materials

The following are available online at https://www.mdpi.com/2077-0383/9/10/3292/s1, Table S1: Test accuracy of RNase 1 and AKI according to the KDIGO classification for all patients suffering from AKI, Table S2: Test accuracy of RNH 1 and AKI according to the KDIGO classification for all patients suffering from AKI, Table S3: Test accuracy of RNase 1 and in-hospital mortality, Table S4: Test accuracy of RNH 1 and in-hospital mortality.
