# Supplementary material for: The Role of Ribonuclease 1 and Ribonuclease Inhibitor 1 in Acute Kidney Injury after Open and Endovascular Thoracoabdominal Aortic Aneurysm Repair
Source: J Clin Med. 2020 Oct 14;9(10):3292. doi: 10.3390/jcm9103292 (PMC7602227; doi:10.3390/jcm9103292)
Supplement: Supplementary file 1 [file jcm-09-03292-s001.pdf]

Journal of Clinical Medicine

Supplementary Materials:

Article

Elisabeth Zechendorf [1,†], Alexander Gombert [2,†], Tanja Bülow [3], Nadine Frank [1], Christian Beckers [1], Arne Peine [1], Drosos Kotelis [2], Michael J. Jacobs [2], Gernot Marx [1] and Lukas Martin [1,*]

[1] Department of Intensive Care and Intermediate Care, University Hospital RWTH Aachen, 52062 Aachen, Germany; ezechendorf@ukaachen.de (E.Z.); nfrank@ukaachen.de (N.F.); cbeckers@ukaachen.de (C.B.); apeine@ukaachen.de (A.P.); gmarx@ukaachen.de (G.M.)
[2] European Vascular Center Aachen-Maastricht, University Hospital RWTH Aachen, 52062 Aachen, Germany; agombert@ukaachen.de (A.G.); dkotelis@ukaachen.de (D.K.); mjacobs@ukaachen.de (M.J.J.)
[3] Department of Medical Statistics, University Hospital RWTH Aachen, 52062 Aachen, Germany; tbuelow@ukaachen.de
* Correspondence: lmartin@ukaachen.de; Tel.: +49-(0)-241-8037606
† Equally contributed authorship.

Table S1. Test accuracy of RNase 1 and AKI according to the KDIGO classification for all patients suffering from AKI. Sensitivity, Specificity and AUC are given with their respective 95 % confidence intervals.

**Time of measurement**	**Optimal Cut-Off (Youden Index)**					**AUC**
	Cut-Off [ng/ml]	Sensitivity [%]	Specificity [%]	LR+	LR-	
0 days	≥ 28.00	86.67 [59.5, 98.3]	50.00 [24.7, 75.3]	1.73	0.27	0.713 [0.523, 0.860]
Admission on ICU	≥ 30.60	92.31 [64.0, 99.8]	43.75 [19.8, 70.1]	1.64	0.18	0.702 [0.504, 0.856]
12 h	≥ 29.19	93.33 [68.1, 99.8]	53.33 [26.6, 78.7]	2.00	0.13	0.742 [0.551, 0.884]
24 h	≥ 32.81	93.33 [68.1, 99.8]	62.50 [35.4, 84.8]	2.49	0.11	0.746 [0.558, 0.884]
48 h	≥ 36.02	100.00 [66.4, 100.0]	56.25 [29.9, 80.2]	2.29	-	0.750 [0.538, 0.900]
72 h	≥ 56.73	84.62 [54.6, 98.1]	61.54 [31.6, 86.1]	2.20	0.25	0.710 [0.500, 0.870]

Table S2. Test accuracy of RNH 1 and AKI according to the KDIGO classification for all patients suffering from AKI. Sensitivity, Specificity and AUC are given with their respective 95 % confidence intervals.

**Time of measurement**	**Optimal Cut-Off (Youden Index)**					**AUC**
	Cut-Off [ng/ml]	Sensitivity [%]	Specificity [%]	LR+	LR-	
0 days	≥ 2.72	66.67 [38.4, 88.2]	68.75 [41.3, 89.0]	2.13	0.48	0.629 [0.438, 0.795]
Admission on ICU	≥ 10.87	85.71 [57.2, 98.2]	81.25 [54.4, 96.0]	4.57	0.18	0.781 [0.593, 0.911]
12 h	≥ 12.39	86.67 [59.5, 98.3]	68.75 [41.3, 89.0]	2.77	0.19	0.788 [0.604, 0.913]
24 h	≥ 9.35	80.00 [51.9, 95.7]	56.25 [29.9, 80.2]	1.83	0.36	0.721 [0.531, 0.866]
48 h	≥ 10.44	71.43 [41.9, 91.6]	75.00 [47.6, 92.7]	2.86	0.38	0.790 [0.603, 0.916]
72 h	≥ 5.43	91.67 [61.5, 99.8]	50.00 [23.0, 77.0]	1.83	0.17	0.702 [0.492, 0.864]

Table S3. Test accuracy of RNase 1 and in-hospital mortality. Sensitivity, Specificity and AUC are given with their respective 95 % confidence intervals.

**Time of measurement**	**Optimal Cut-Off (Youden Index)**					**AUC**
	Cut-Off [ng/ml]	Sensitivity [%]	Specificity [%]	LR+	LR-	
0 days	≥ 67.70	50.00 [11.8, 88.2]	81.48 [61.9, 93.7]	2.70	0.61	0.642 [0.457, 0.801]
Admission on ICU	≥ 41.36	100.00 [47.8, 100.0]	61.54 [40.6, 79.8]	2.60	-	0.769 [0.584, 0.901]
12 h	≥ 67.36	83.33 [35.9, 99.6]	84.62 [62.1, 95.6]	5.42	0.20	0.821 [0.645, 0.933]
24 h	≥ 64.28	60.00 [14.7, 94.7]	81.48 [61.9, 93.7]	3.24	0.49	0.659 [0.471, 0.817]
48 h	≥ 55.58	100.00 [29.2, 100.0]	59.26 [38.8, 77.6]	2.45	-	0.790 [0.603, 0.916]
72 h	≥ 122. 67	100.00 [15.8, 100.0]	91.67 [73.0, 99.0]	12.0	-	0.938 [0.769, 0.995]

Table S4. Test accuracy of RNH 1 and in-hospital mortality. Sensitivity, Specificity and AUC are given with their respective 95 % confidence intervals.

**Time of measurement**	**Optimal Cut-Off (Youden Index)**					**AUC**
	Cut-Off [ng/ml]	Sensitivity [%]	Specificity [%]	LR+	LR-	
0 days	≥ 9.89	66.67 [22.3, 95.7]	85.19 [66.3, 95.8]	4.50	0.56	0.710 [0.526, 0.854]
Admission on ICU	≥ 10.87	80.00 [28.4, 99.5]	51.85 [31.9, 71.3]	1.66	0.39	0.526 [0.343, 0.704]
12 h	≥ 16.09	83.33 [35.9, 99.6]	66.67 [46.0, 83.5]	2.50	0.25	0.735 [0.553, 0.872]
24 h	≥ 19.84	50.00 [6.8, 93.2]	88.89 [70.8, 97.6]	4.50	0.56	0.565 [0.376, 0.741]
48 h	≥ 16.44	66.67 [9.4, 99.2]	88.89 [70.8, 97.6]	6.00	0.38	0.679 [0.484, 0.837]
72 h	≥ 16.41	50.00 [1.3, 98.7]	95.83 [78.9, 99.9]	12.00	0.52	0.542 [0.337, 0.737]
